# Prevalence and Clinical Features of Hearing Loss Patients with *CDH23* Mutations: A Large Cohort Study

**DOI:** 10.1371/journal.pone.0040366

**Published:** 2012-08-10

**Authors:** Maiko Miyagawa, Shin-ya Nishio, Shin-ichi Usami

**Affiliations:** Department of Otorhinolaryngology, Shinshu University School of Medicine, Matsumoto, Japan; Stanford University School of Medicine, United States of America

## Abstract

Screening for gene mutations in *CDH23*, which has many exons, has lagged even though it is likely to be an important cause for hearing loss patients. To assess the importance of *CDH23* mutations in non-syndromic hearing loss, two-step screening was applied and clinical characteristics of the patients with *CDH23* mutations were examined in this study. As a first screening, we performed Sanger sequencing using 304 probands compatible with recessive inheritance to find the pathologic mutations. Twenty-six possible mutations were detected to be pathologic in the first screening. For the second screening, using the probes for these 26 mutations, a large cohort of probands (n = 1396) was screened using Taqman amplification-based mutation analysis followed by Sanger sequencing. The hearing loss in a total of 52 families (10 homozygous, 13 compound heterogygous, and 29 heterozygous) was found to be caused by the *CDH23* mutations. The majority of the patients showed congenital, high frequency involved, progressive hearing loss. Interestingly, some particular mutations cause late onset moderate hearing loss. The present study is the first to demonstrate the prevalence of *CDH23* mutations among non-syndromic hearing loss patients and indicated that mutations of the *CDH23* gene are an important cause of non-syndromic hearing loss.

## Introduction

Mutations in the *CDH23* (NM_22124) gene are known to be responsible for both Usher syndrome type ID (USH1D) and non-syndromic hearing loss (DFNB12) [1], [2]. Molecular confirmation of *CDH23* mutations has become important in the diagnosis of these conditions.

This gene encodes cadherin 23, a protein of 3354 amino acids with 27 extracellular (EC) domains, a single transmembrane domain and a short cytoplasmic domain. Cadherin-specific amino acid motifs such as DRE, DXNDN, and DXD, that are highly conserved in sequence and spacing and required for cadherin dimerization and calcium binding were found in each extracelluar domain [3].

The cadherin 23 protein is known to be an important composition of the tip link that maintains the arrangement of streocilia [4].

More than 50 mutations have been reported for the Usher phenotype (USH1D) and 24 mutations reported for the non-syndromic hearing loss phenotype (DFNB12) [1], [2], [5]–[7]. As suggested by genotype–phenotype correlation study, Usher 1D, which has congenital profound hearing impairment, vestibular dysfunction, and retinitis pigmentosa, is usually associated with nonsense mutations, whereas DFNB12, which has a milder phenotype, is associated with missense mutations [1], [2], [5]–[8].

We previously reported that four pathologic mutations were identified in 5 out of 64 Japanese families compatible with autosomal recessive inheritance, suggesting that *CDH23*-caused deafness may be commonly found among non-syndromic hearing loss patients [6]. *GJB2* has been shown to be a common gene involved in congenital hearing impairment. *SLC26A4* is also frequently involved among those patients. *GJB2* and *SLC26A4* are comparatively small making Sanger sequencing relatively easy. The latter is also associated with the typical inner ear anomaly, enlarged vestibular aqueduct. Therefore, screening is relatively easy and many studies have focused on just these two genes. Clinical molecular diagnosis has been dramatically improved for these genes. However, screening strategy of other hearing loss genes is difficult and Sanger sequencing of the candidate genes, such as *CDH23*, with many exons is time consuming. Consequently, only a few reports are available for the mutation spectrum of *CDH23*.

In the present study, we performed Sanger sequencing using 304 patients whose pedigrees are compatible with recessive inheritance to find additional pathologic mutations. Also, to find the novel pathologic mutations and to clarify the frequency and clinical characteristics of patients with *CDH23* mutations, a large cohort of probands from unrelated families (n = 1396) was screened using TaqMan amplification-based mutation analysis of the variants observed in the initial 304 patients.

## Results

The first screening using 304 Japanese probands compatible with autosomal recessive inheritance identified 26 candidates for disease causing mutations. These include four previously reported pathologic mutations: p.P240L, p.R301Q, p.Q1716P, and p.R2029W, as well as 6 possible pathologic variants in the coding region of *CDH23*. All of the mutations were missense mutations. The following second screening based on TaqMan assay followed by Sanger sequencing confirmed 10 “possibly pathologic” mutations (Table 1) and 17 variants with uncertain pathogenicity (Table 2) in a large cohort of the patients. “Possible pathologic” mutations were defined as 1) mutations found to be homozygotes or compound heterozygotes (and determined by segregation study), 2) variants which were not found or were very few in 192 control subjects, 3) amino acids that were well-conserved among various species, 4) compatible with next generation sequencing database, and 5) compatible with the predicted effect of missense mutations on *CDH23* protein function. Results of the compatibility of the next generation sequence database, the SIFT and PolyPhen2 score for prediction are shown in Tables 1 and 2.

**Table 1:** Possible pathologic variants found in this study.

Amino acid change	Nucleotide change	EXON	Domain	Evolutionary conservation	The highly conserved calcium-binding elements	Number in probands (n = 1396)			Allele frequency in patients (in 2792 allele)	Allele frequency in control (in 384 allele)	Allele frequency in HL patients based on a Next generation sequencing database (in 432 allele)	Allele frequency in controls based on a Next generation sequencing database (in 144 allele)	PolyPhen 2 score*	SIFT Score*	Reference
						homozygote	compound heterozygote	heterozygote							
p.P240L	c.719C>T	7	EC3	7	-	7	12	19	1.612	0.260	0.63	0.67	0.999	0.06	Wagatsuma et al.
p.R301Q	c.902G>A	9	EC3	7	DRE	-	3	-	0.107	0.260	0	0	1.000	0	Wagatsuma et al.
p.E956K	c.2866G>A	25	EC9	7	DRE	-	1	2	0.107	0	0.21	0	1.000	0.04	this study
p.T1368M	c.4103C>T	32	EC13	7	-	-	1	-	0.036	0	0	0	1.000	0	this study
p.R1417W	c.4249C>T	35	EC13	5	-	1	-	2	0.143	0	0.25	0	0.998	0.19	Wagatsuma et al.
p.D1626A	c.4877A>C	39	EC15	7	DXNDN	-	1	-	0.036	0	0	0	0.999	0.01	this study
p.Q1716P	c.5147A>C	39	EC16	7	-	-	3	-	0.107	0	0	0	0.957	0.3	Wagatsuma et al.
p.R2029W	c.6085C>T	46	EC19	7	DRE	2	2	6	0.430	0	0	0	0.999	0.01	Wagatsuma et al.
p.N2287K	c.6861T>G	50	EC21	7	DXNDN	-	2	-	0.072	0	0	0	0.971	0	this study
p.E2438K	c.7312G>A	52	EC23	6	-	-	1	-	0.036	0	0	0	0.986	1	this study

* Computer analysis to predict the effect of missense variants on *CDH23* protein function was performed with Sorting Intolerant from Tolerant (SIFT; http://sift.jcvi.org/), and Polymorphism Phenotyping (PolyPhen2;http://genetics.bwh.harvard.edu/pph2/).

**Table 2:** Variants with uncertain pathogenicity found in this study.

Amino acid change	Nucleotide change	EXON	Domain	Evolutionary conservation	The highly conserved calcium-binding elements	Number in probands (n = 1396)			Allele frequency in patients (in 2792 allele)	Allele frequency in control (in 384 allele)	Allele frequency in HL patients based on a Next generation sequencing database (in 432 allele)	Allele frequency in controls based on a Next generation sequencing database (in 144 allele)	PolyPhen 2 score***	SIFT Score***	Reference
						homozygote	compound heterozygote	heterozygote							
p.D160N	c.478G>A	4	EC2	7	DXD	-	-	2	0.072	0.260	0	0	1.000	0	this study
p.V803I	c.2407G>A	23	EC8	7	-	-	-	3	0.107	0	0	0	0.761	0.41	this study
p.S1415I	c.4244G>T	35	EC13	7	-	-	-	1	0.036	0	0	0	0.840	0.06	this study
p.A1443G *	c.4328C>G	35	EC14	7	-	1*	-	2	0.143	0	0.2	0	0.944	0.06	this study
p.R1588W **	c.4762C>T	38	EC15	7	-	4**	-	18	0.931	0.260	2.22	0	1.000	0.01	Wagatsuma et al.
p.V1711I	c.5131G>A	40	EC16	7	-	-	-	2	0.072	0	0	0	0.970	0.12	Wagatsuma et al.
p.V1807M	c.5419G>A	42	EC17	5	-	-	1	-	N/A	0.260	0	0	0.054	0.22	this study
p.S1876N	c.5627G>A	43	EC18	5	-	-	-	6	0.215	0	0	0	0.981	0.26	Wagatsuma et al.
p.V1908I	c.5722G>A	44	EC9	5	-	-	-	12	0.430	0.260	1.09	0.53	0.948	1	Wagatsuma et al.
p.A2130V	c.6389C>T	48	EC20	6	-	-	-	1	0.036	0	0	0	0.999	0.24	this study
p.R2171C	c.6511C>T	48	EC20	7	DXNDNR	-	-	1	0.036	0.521	0	0	0.999	0.11	Wagatsuma et al.
p.Q2227P	c.6680A>C	48	EC21	6	-	-	-	1	0.036	0.260	0	0	0.930	0.2	Wagatsuma et al.
p.L2473P	c.7418T>C	53	EC23	7	-	-	-	1	0.036	0	0	0	0.999	0	Wagatsuma et al.
p.I2669V	c.8005A>G	56	EC25	5	-	-	-	1	0.036	0	0	0	0.134	0.7	Wagatsuma et al.
p.F2801V	c.8401T>G	59	EC26	5	-	-	-	1	0.036	0.781	1.52	1.27	0.800	0.01	Wagatsuma et al.
p.G2912S	c.8734G>A	61	EC27	7	-	-	-	1	0.036	0	0.23	0	0.996	0	this study
p.R3175C	c.9523C>T	68	CYTO	7	-	-	-	1	0.036	0.260	0	0	0.886	0.01	Wagatsuma et al.

* not confirmed by segregation study.

** one normal hearing subject with homozygotes.

*** Computer analysis to predict the effect of missense variants on *CDH23* protein function was performed with Sorting Intolerant from Tolerant (SIFT; http://sift.jcvi.org/), and Polymorphism Phenotyping (PolyPhen2;http://genetics.bwh.harvard.edu/pph2/).

N/A: TaqMan probe not available.

The 17 variants found as heterozygous and therefore with uncertain pathogenicity did not fulfill all the above criteria. For example, p.A1443G was uncertain because DNA samples from family members were not available and we could not confirm its pathogenicity by segregation study. p.R1588W was found to be homozygous in 4 patients and heterozygous in 16 patients, but only 1 was found in 384 control alleles. However, a member of the patient's family (#2841) showed normal hearing instead of being homozygous. Also p.V803I, p.V1807M and p.I2669V are obscure from the functional prediction analysis.

In one family (#4685), three mutation were found in proband and two of them were found in same allele p.[D16126A;V1807M] confirmed by segregation analysis.

As p.V1807M predicted to have no effect on *CDH23* structure, p.D1626A might be a pathogenic mutation.

**Figure 1:**
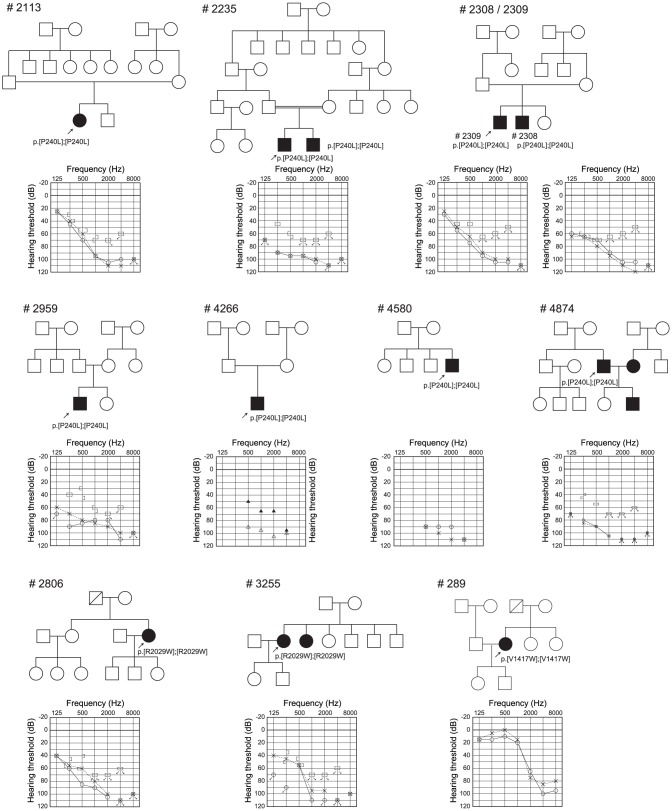
Pedigrees, mutations, and audiograms of the patients with homozygous *CDH23* mutations.

**Figure 2:**
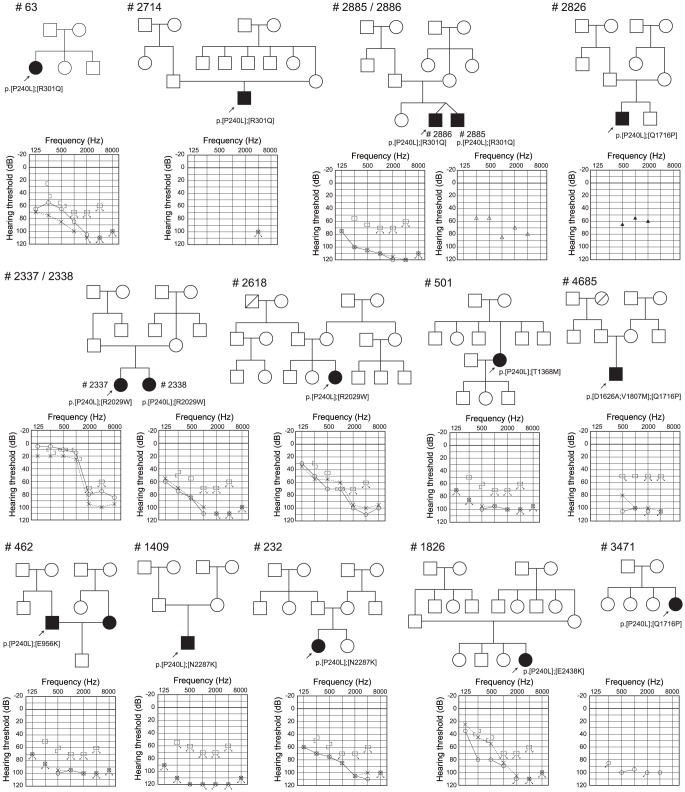
Pedigrees, mutations, and audiograms of the patients with compound heterozygous *CDH23* mutations.

For 10 possible pathologic mutations, amino acids were well-conserved among various species, including *Homo sapiens, P. troglodytes, B. traurus, M. musculus, R. norvegicus, G. gallus*, and *D. rario*. Many mutations (5 out of 10 possible pathologic mutations, 2 out of 17 uncertain variants) were found in DRE, DXNDN, and DXD motif (Table 1 and 2). Ten possible pathologic mutations were found to be either homozygotes (n = 11, Table 3, Fig. 1) or compound heterozygotes (n = 15) (Table 4, Fig. 2). Twenty-nine patients were found to be heterozygous without a second mutation (Table 5).

**Table 3:** Details of phenotype and genotype of 11 patients in 10 families with homozygous *CDH23* mutation.

Sample No	relationship	Amino acid Change	Hereditary form	Threshold* (Rt)(dB)	Threshold* (Lt)(dB)	severity	Residual hearing in the lower frequencies** (dB)	Hearing in the higher frequencies*** (dB)	Age	Age of awareness	Progressiveness	Hearing aid/cochlear implant	Vertigo	Tinnitus
#2113		p.[P240L];[P240L]	sporadic	91.3	90	severe	44.2	104.2	12	6	+	HA	−	−
#2235		p.[P240L];[P240L]	AR	97.5	96.3	profound	85.0	104.2	22	0	−	HA	−	−
#2308		p.[P240L];[P240L]	AR	88.8	95	severe	67.5	110.0	11	0****	−	HA	−	−
#2309	sibling of #2308	p.[P240L];[P240L]	AR	92.5	86.3	severe	50.0	105.0	9	0****	−	HA	−	−
#2959		p.[P240L];[P240L]	sporadic	81.3	85	severe	75.8	96.7	8	0****	−	HA	−	−
#4266		p.[P240L];[P240L]	sporadic	96.3	96.3	severe	70.0	91.3	3	0****	+	CI	−	−
#4580		p.[P240L];[P240L]	sporadic	102.5	97.5	profound	88.3	106.7	1	0****	−	CI	−	N/A
#4874		p.[P240L];[P240L]	sporadic	102.5	102.5	profound	80.8	106.7	38	2	+	HA	−	−
#2806		p.[R2029W];[R2029W]	sporadic	92.5	80	severe	56.7	104.2	53	48	+	HA	−	+
#3255		p.[R2029W];[R2029W]	AR	96.3	85	severe	59.2	104.2	71	60	+	HA	−	+
#289		p.[V1417W];[V1417W]	sporadic	31.3	26.3	mild	10.0	85.0	34	14	+	HA	−	−

* average of 500, 1000, 2000 and 4000 Hz.

** average of 125, 250, and 500 Hz.

*** average of 2000, 4000, and 8000 Hz.

**** found by newborn hearing screening.

**Table 4:** Details of phenotype and genotype of 15 patients in 13 families with compound heterozygous *CDH23* mutation.

Sample No	relationship	Amino acid Change	Hereditary form	Threshold* (Rt)(dB)	Threshold* (Lt)(dB)	severity	Residual hearing in the lower frequencies** (dB)	Hearing in the higher frequencies*** (dB)	Age	Age of awareness	Progressiveness	Hearing aid/cochlear implant	Vertigo	Tinnitus
#63		p.[P240L];[R301Q]	sporadic	85	98.8	severe	69.2	105.8	27	0	−	HA	−	+
#2714		p.[P240L];[R301Q]	sporadic	97.5	97.5	profound	71.7	105.0	2	0****	+	HA	−	−
#2885		p.[P240L];[R301Q]	AR	90	108.7	profound	55.0	75.0	13	3	+	CI	−	−
#2886	sibling of #2885	p.[P240L];[R301Q]	AR	115	110	profound	93.3	115.8	13	2	+	CI	−	−
#2337		p.[P240L];[R2029W]	AR	30	41.3	mild	13.3	88.3	13	11	+	HA	−	+
#2338	sibling of #2337	p.[P240L];[R2029W]	AR	103.8	98.8	profound	71.7	106.7	8	2	+	HA	−	−
#2618		p.[P240L];[R2029W]	sporadic	77.5	67.5	moderate	49.2	100.0	8	3	+	CI	−	−
#2826		p.[P240L];[Q1716P]	sporadic	91.3	95	profound	66.7	112.5	6	0	+	HA	−	−
#3471		p.[P240L];[Q1716P]	sporadic	97.5	97.5	profound	92.5	100.0	4	0	−	CI	−	−
#462		p.[P240L];[E956K]	sporadic	97.5	97.3	profound	84.2	98.3	38	10	−	HA	−	−
#501		p.[P240L];[T1368M]	sporadic	>90	>90	profound	N/A	N/A	68	44	+	HA	+	+
#1409		p.[P240L];[N2287K]	sporadic	120	120	profound	107.5	123.3	17	0	+	HA	−	−
#232		p.[P240L];[N2287K]	sporadic	87.5	86.3	severe	67.5	104.2	15	0	−	HA	−	+
#1826		p.[P240L];[E2438K]	sporadic	91.3	106.3	severe	70.8	105.8	11	3	+	HA	−	−
#4685		p.[D1626A;V1807M];[Q1716P]	sporadic	97.5	103.8	severe	96.3	105.0	1	0*	−	CI	−	N/A

* average of 500, 1000, 2000 and 4000 Hz.

** average of 125, 250, and 500 Hz.

*** average of 2000, 4000, and 8000 Hz.

**** found by newborn hearing screening.

**Table 5:** Details of phenotype and genotype of 29 patients with heterozygous *CDH23* mutation.

Sample No	relationship	Amino acid Change	Hereditary form	Threshold* (Rt)(dB)	Threshold* (Lt)(dB)	severity	Residual hearing in the lower frequencies** (dB)	Hearing in the higher frequencies*** (dB)	Age	Age of awareness	Progressiveness	Hearing aid/cochlear implant	Vertigo	Tinnitus
#334		p.[P240L];[-]	AD	96.25	83.75	severe	63.3	96.7	23	0	+	HA	N/A	+
#340		p.[P240L];[-]	sporadic	>90	>90	profound	N/A	N/A	54	14	+	HA	N/A	N/A
#569		p.[P240L];[-]	sporadic	86.25	90	severe	75.0	98.3	26	3	+	HA	−	−
#653		p.[P240L];[-]	sporadic	53.75	57.5	moderate	44.2	71.7	36	33	+	HA	−	+
#754		p.[P240L];[-]	sporadic	110	101.25	profound	87.5	104.2	57	0	+	HA	N/A	N/A
#1039		p.[P240L];[-]	sporadic	48.75	56.25	moderate	33.3	74.2	76	76	−	HA	+	−
#1598		p.[P240L];[-]	sporadic	56.25	10	unilateral	34.2	41.7	60	49	−	−	+	+
#1807		p.[P240L];[-]	sporadic	110	8.75	unilateral	50.8	60.0	50	9	−	−	−	−
#1846		p.[P240L];[-]	AD	100	96.25	profound	83.3	98.3	62	6	+	HA	+	+
#2159		p.[P240L];[-]	AR	67.5	66.25	moderate	60.0	69.2	10	65	+	HA	−	−
#2374		p.[P240L];[-]	AR	86.25	90	severe	78.3	78.3	5	0	−	HA	−	−
#2835		p.[P240L];[-]	sporadic	85	91.25	severe	65.8	101.7	12	3	+	HA	+	−
#3492		p.[P240L];[-]	AD	103.75	103.75	profound	88.8	107.5	1	0	−	HA	−	−
#3499		p.[P240L];[-]	AD	96.25	110	severe	84.2	105.8	57	50	−	CI	−	+
#3761		p.[P240L];[-]	AR	32.5	40	mild	43.3	75.8	71	0	−	−	−	+
#4040		p.[P240L];[-]	AR	S/O	S/O	profound	S/O	S/O	2	0	+	HA	−	−
#4159		p.[P240L];[-]	AR	97.5	71.25	severe	71.7	95.0	38	38	+	HA	+	+
#4313		p.[P240L];[-]	AD/Mit	130	102.5	profound	107.5	116.7	6	0	−	CI	−	−
#4615		p.[P240L];[-]	sporadic	90	90	profound	90.0	90.0	0	0****	−	CI	−	−
#265		p.[E956K];[-]	sporadic	110	6.25	unilateral	57.5	59.2	16	0	−	−	−	−
#3116		p.[E956K];[-]	AD	47.5	53.75	moderate	58.3	40.8	63	N/A	+	HA	−	+
#280		p.[R1417W];[-]	sporadic	110	6.25	unilateral	50.0	55.8	8	3	−	−	N/A	N/A
#2649		p.[R1417W];[-]	sporadic	95	110	profound	87.5	105.0	11	0	+	CI	−	N/A
#1131		p.[R2029W];[-]	sporadic	73.75	72.5	severe	55.0	93.3	24	17	+	HA	−	−
#1539		p.[R2029W];[-]	AD	53.75	110	moderate	70.0	83.3	71	60	+	HA	−	+
#1618		p.[R2029W];[-]	sporadic	26.25	61.25	mild	31.7	60.8	67	N/A	−	−	−	+
#1919		p.[R2029W];[-]	AD	38.75	36.25	mild	20.8	75.0	25	3	+	−	N/A	N/A
#2271		p.[R2029W];[-]	AD	58.75	62.5	moderate	41.7	50.0	6	N/A	N/A	HA	N/A	N/A
#4138		p.[R2029W];[-]	AR	71.25	53.75	moderate	50.8	65.8	10	3	+	HA	+	−

* average of 500, 1000, 2000 and 4000 Hz.

** average of 125, 250, and 500 Hz.

*** average of 2000, 4000, and 8000 Hz.

**** found by newborn hearing screening.

**Figure 3:**
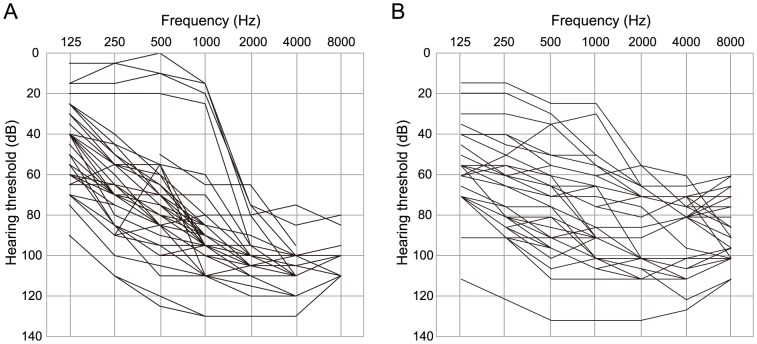
Overlapping audiograms of the patients with *CDH 23* mutations. A: patients with hearing loss caused by the *CDH23* mutations (homozygous or compound heterozygous cases), B: patients potentially caused by the *CDH23* mutations (heterozygous cases).

**Figure 4:**
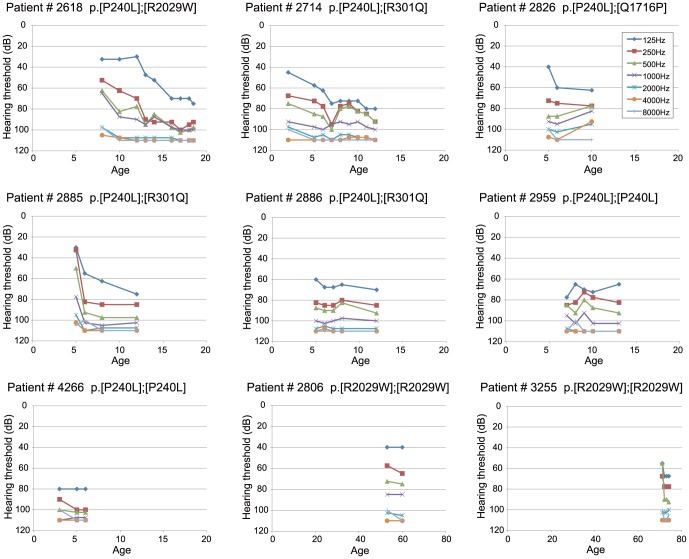
Hearing progression of the patients with *CDH23* mutations. Note that the high frequency portion was already worsened, and the low frequency portion was deteriorated by ages.

Tables 3 and 4 summarize 23 families with hearing loss caused by the *CDH23* mutations (homozygous or compound heterozygous cases) and Table 5 summarizes 29 families with hearing loss potentially caused by the *CDH23* mutations (heterozygous cases). The frequency was 1.6% (23/1396) or 2.1% (29/1396) of the overall hearing loss population. When restricted to patients compatible with recessive inheritance, the frequency was increased to 2.5% (23/919) or 3.2% (29/919). Table 3, 4 and 5 also summarize clinical characteristics including hereditary form, hearing threshold, severity, residual hearing in the lower frequencies, hearing in the higher frequencies, onset age (age of awareness), progressiveness of hearing loss, use of hearing aid/cochlear implantation, visual impairment, and vestibular symptoms. The ages of these patients were from 1 to 71 years. Age of onset (awareness of hearing loss) ranged from congenital to 60 years old, though the majority was congenital or early onset. There were some correlations between genotype and phenotype (onset age). The patients associated with p.P240L showed congenital and severe hearing loss regardless of whether associated with one more mutation, whereas the patients with p.R2029W or p.T1368M showed late-onset moderate hearing loss (Tables 3 and 4). Concerning type of hearing loss, the majority of the patients had some residual hearing in the lower frequencies, and overlapping audiograms showed characteristic high frequency involved hearing loss (Fig. 3). The majority of the patients showed progressive nature of hearing loss evaluated by serial audiogram (Fig. 4). No patients had associated visual impairment or vestibular symptoms (Tables 3, 4 and 5). Seven patients received cochlear implantation due to the insufficient amplification of hearing aids (Tables 3, 4 and 5).

## Discussion

Mutations in the *CDH23* gene are known to be responsible for both Usher syndrome type ID (USH1D) as well as non-syndromic hearing loss (DFNB12), and molecular confirmation of *CDH23* mutations is clinically important for diagnosis of these conditions. However, clinical application of the detection of *CDH23* mutations has lagged because of the size of the gene. Especially for DFNB12, which is not associated with visual impairment, screening is comparatively difficult, and therefore, little is known about frequencies among the hearing loss population as well as clinical characteristics.

In this study, we have applied two-step screening and identified a significant number of novel pathologic mutations of *CDH23* responsible for non-syndromic hearing loss in a large cohort of patients. All of the possible pathologic mutations identified in this study (Table 1) were missense mutations, being consistent with previous reports that DFNB12 patients associated with missense mutations have milder hearing impairment than in USH1D, which is associated with nonsense, splice-site, or frameshift mutations [2], [5]–[7]. None had visual impairment, also supporting this rule. That the majority was found in the EC domain with only one exception found in the cytoplasmic domain, was also in line with the previous reports on DFNB12 [2], [5]–[7]. Of these 26 mutations, five out of 10 possible pathologic mutations were found in DRE, DXNDN, and DXD motifs, which are thought to be important for calcium binding property. These highly conserved EC calcium binding motifs are thought to be essential for linearization, rigidification, and dimerization of the cadherin molecules [9], [10]. And the results of computer analysis to predict the impact of amino acid change, all of 10 possible pathologic mutations predicted to cause a severe damage for protein function of *CDH23*.

As a result, 26 patients (from 23 families) had two mutations (in a homozygous or compound heterozygous state), and met criteria for recessive inheritance. A hallmark of recessive mutations is the detection of two mutations in the paternal and maternal alleles and the parents having normal hearing. As seen in previous mutation screening reports, including those for *CDH23* [6], [7] as well as *GJB2* and *SLC26A4* [11], [12], we encountered a significant number of heterozygous cases without a second mutation even after direct sequencing of the coding region of the gene. Possible explanations are: 1) the existence of a second mutation in the intron or regulatory region of *CDH23*, which has not been explored, 2) the observed mutations are rare polymorphisms, 3) the screening method fails to detect the second mutation, and 4) an additional modulatory gene may contribute to hearing loss (for example, *PCDH15*). Although we have not reached the final conclusion, it is most likely that these heterozygous cases are also related to *CDH23* mutations because: 1) allele frequencies are found to be higher in the hearing loss group (Table 2), and 2) the phenotype is similar to that of the patients with two mutations. As shown in Fig. 3, overlapping audiograms of the patients with only one mutation was similar to that with the patients with two mutations (high frequency involved sensorineural hearing loss with residual hearing at the lower frequencies).

Based on the frequencies of 3.7% (including heterozygous cases) of the hearing loss population and 5.7% (including heterozygous cases) of the recessive inherited cases in this study, we confirmed that mutations of *CDH23* are an important cause for non-syndromic hearing loss and should be borne in mind next to *GJB2* or *SLC26A4* screening. This study revealed that p.P240L account for nearly 43.3%(45/104) of all *CDH23* mutated families in Japan. Common mutations, such as c.35delG or c.235delC in *GJB2* or p.H723R in the *SLC26A4* gene, have been reported in many recessive deafness genes, and usually they are population-specific [12]–[14]. It is an interesting question whether p.P240L is frequent because of a founder effect or mutational hot spot, but the existence of such a common mutation makes mutation screening easier. Additional frequent mutations found in this study together with TaqMan procedures will facilitate genetic testing for deafness patients.

Concerning mutation spectrum, as in our previous report [6], the *CDH23* mutation spectrum in Japanese is very different from that found in Caucasians and may be representative of those in Eastern Asian populations. Its elucidation is expected to facilitate the molecular diagnosis of DFNB12 and USH1D. It has also been known that prevalent *GJB2* mutations are highly ethnic-specific (see The connexin-deafness homepage; http://davinci.crg.es/deafness/): c.35delG is common in the Caucasoid population, c.167delT was reported as prevalent in Ashkenazi Jews, p.R143W in a restricted village in Africa, and c.235delC in East Asian populations. A series of studies proved a founder effect for these frequent mutations [11], [15].

In the present study, using a large cohort of patients, clinical characteristics (onset age, progression, audiograms) of patients with *CDH23* mutations were clarified.

Concerning genotype/phenotype correlations, hearing of the patients with p.[P240L];[P240L] is worse than in those with the other mutations, and tends to be congenital and severe. In contrast, the patients with p.[R2029W];[R2029W] showed a milder phenotype of middle age onset. Overlapping audiograms showed typical high frequency involved sensorineural hearing loss with residual hearing at the lower frequencies.

Concerning age of onset (awareness of hearing loss), the majority was congenital or early onset. But rather later-onset was seen in three patients (#2806, 3255, 501), and they were associated with some particular mutations (p.R2029W and p.T1368M). Their phenotype was rather mild and gradually progressive. It is interesting to note that their phenotype was similar to presbycusis. Actually, *CDH23* mutations have been reported as responsible for age-related hearing loss in mice [16], [17].

Progressive nature of hearing loss and the presence of residual hearing are particular phenotypic features of the patients with *CDH23* mutations. Our previous genetic analysis for the patients with high frequency involved hearing loss successfully identified *CDH23* mutations [18]. Seven patients received cochlear implantation and showed good performance after implantation. For the patients with residual hearing, newly developed cochlear implantation; EAS (Electric Acoustic Stimulation) is a good therapeutic option and therefore much attention should be paid to the etiology when considering individual intervention, i.e., regular cochlear implantation or EAS. Genetic testing will be very important prognostic information together with various hearing tests.

In conclusion, a large cohort study using Taqman amplification-based mutation analysis indicated that mutations of the *CDH23* gene are important causes of non-syndromic hearing loss. A mutation screening strategy using TaqMan assay based on the ethnic-specific frequent mutations is a powerful and effective method for such a large gene. Clinical characteristics of patients with *CDH23* mutations is that hearing loss is progressive, high frequency involved sensorineural hearing loss with residual hearing in the lower frequencies. Most cases are congenital but care is needed because some patients show presbycusis-like hearing loss. Cochlear implantation (including EAS) is a good therapeutic intervention for the patients with *CDH2*3 mutations.

## Materials and Methods

To identify additional pathologic *CDH23* mutations, two-step screening was applied in this study. Subjects from independent families were collected from 33 ENT departments nationwide in Japan. All subjects gave prior informed consent for participation in the project, which was approved by the ethical committee of each hospital. Genomic DNA was isolated from peripheral blood by DNeasy Blood and Tissue Kit (QIAGEN, Düsseldorf, Germany) according to the manufacturer's procedure.

### First screening (Direct sequencing)

First, we sequenced the *CDH23* gene in 304 Japanese non-syndromic sensorineural hearing loss probands (including our previously reported 64 samples [6]) compatible with autosomal recessive inheritance or sporadic cases. None of the subjects had any other associated neurological signs, vestibular or visual dysfunction. Sanger sequencing was applied to these samples to find mutations responsible for deafness. Detailed procedures were described in our previous report [6]. 26 candidates for disease causing mutations were collected according to the following criteria; 1) non-synonymous variants, and 2) allele carrier rates were less than 2% in control subjects.

### Second screening (TaqMan genotyping assay based screening and Direct sequencing)

For the second screening, probes of these 26 mutations selected in the first screening was applied for a custom TaqMan® SNP Genotyping Assays (Applied Biosystems, Foster City, CA) [19]. 1396 probands of sensorineural hearing loss patients including 304 probands used in the first screening were used for the second assay. Of them, 1347 had bilateral sensorineural hearing loss and 49 had unilateral sensorineural hearing loss. The inheritance composition of the subjects was as follows: 298 subjects from autosomal dominant or maternally inherited families (two or more generations affected); 919 subjects from autosomal recessive families (parents with normal hearing and two or more affected siblings) or subjects with sporadic deafness (compatible with recessive inheritance or non-genetic hearing loss); the rest had unknown inheritance mode. After TaqMan assay, Sanger sequencing was performed: 1) to confirm these mutations found in TaqMan genotyping assays, 2) to confirm whether mutations were homozygotes or heterozygote, and 3) in cases found in heterozygous state, direct sequencing of the coding region of the *CDH23* was performed.

### Controls

The control group consisted of 192 unrelated Japanese individuals without any noticeable hearing loss evaluated by auditory testing.

### Next generation sequencing and computer analysis

To elucidate the allele frequency of 26 mutations, comparison was made between allele frequency found in 216 deafness patients and 72 controls based on a next generation sequencing database that is currently being established at Shinshu University (unpublished). In brief, exome sequencing was performed with SureSelect target DNA enrichment (Agilent Technologies, Santa Clara, CA) and Illumina GAIIx sequencing (Illumina, San Diego, CA) according to the manufacturers' procedures. In the SureSelect library, 76 already reported genes responsible for sensorineural hearing loss and syndromic hearing loss were contained. After base calling, sequence results were aligned with a bowtie program [20] and allele frequencies of each *CDH23* mutation in patients and the control population were calculated. Computer analysis to predict the effect of missense variants on *CDH23* protein function was performed with Sorting Intolerant from Tolerant (SIFT; http://sift.jcvi.org/), and Polymorphism Phenotyping (PolyPhen2; http://genetics.bwh.harvard.edu/pph2/) [21], [22].
